# Pronounced somatic bottleneck in mitochondrial DNA of human hair

**DOI:** 10.1098/rstb.2019.0175

**Published:** 2019-12-02

**Authors:** Alison Barrett, Barbara Arbeithuber, Arslan Zaidi, Peter Wilton, Ian M. Paul, Rasmus Nielsen, Kateryna D. Makova

**Affiliations:** 1 Department of Biology, Penn State University, University Park, PA, USA; 2 Department of Integrative Biology, University of California at Berkeley, Berkeley, CA, USA; 3 Department of Pediatrics, Penn State College of Medicine, Hershey, PA, USA

**Keywords:** mitochondrion, mtDNA, heteroplasmy, somatic bottleneck, hair development, forensics

## Abstract

*Heteroplasmy* is the presence of variable mitochondrial DNA (mtDNA) within the same individual. The dynamics of heteroplasmy allele frequency among tissues of the human body is not well understood. Here, we measured allele frequency at heteroplasmic sites in two to eight hairs from each of 11 humans using next-generation sequencing. We observed a high variance in heteroplasmic allele frequency among separate hairs from the same individual—much higher than that for blood and cheek tissues. Our population genetic modelling estimated the somatic bottleneck during embryonic follicle development of separate hairs to be only 11.06 (95% confidence interval 0.6–34.0) mtDNA segregating units. This bottleneck is much more drastic than somatic bottlenecks for blood and cheek tissues (136 and 458 units, respectively), as well as more drastic than, or comparable to, the germline bottleneck (equal to 25–32 or 7–10 units, depending on the study). We demonstrated that hair undergoes additional genetic drift before and after the divergence of mtDNA lineages of individual hair follicles. Additionally, we showed a positive correlation between donor's age and variance in heteroplasmy allele frequency in hair. These findings have important implications for forensics and for our understanding of mtDNA dynamics in the human body.

This article is part of the theme issue ‘Linking the mitochondrial genotype to phenotype: a complex endeavour’.

## 1. Background

Mitochondria play an essential role in cellular energy metabolism via coding for important genes involved in bioenergetics [1,2]. The mammalian mitochondrial DNA (mtDNA) is inherited through the maternal lineage. In humans, mtDNA is a circular molecule composed of 16 569 base pairs and containing 37 genes [1]. mtDNA has an elevated mutation rate compared to nuclear DNA probably because of an increased number of replications per cell division, a less efficient DNA repair system and/or exposure to oxygen radicals generated as a by-product of oxidative phosphorylation [3]. Each cell has hundreds to thousands of mtDNA copies that replicate continuously in both proliferating and differentiated cells, which might lead to a rapid accumulation of somatic mutations [4]. mtDNA mutations may result in mitochondrial diseases [1,5] and have also been linked to ageing [6].

The population of mtDNA within the same individual, tissue, cell or even the same mitochondrion can be genetically heterogeneous, a phenomenon known as *heteroplasmy* [1]. Heteroplasmy may result from a *de novo* mutation or through inheritance of existing variants via the maternal lineage [1]. Heteroplasmy at a site can vary in allele frequencies between cells, tissues or organs of an individual [1]. Many mtDNA diseases are heteroplasmic, and a certain allele frequency for a disease-causing variant must be reached before disease symptoms become apparent [1,2]. Heteroplasmy has also been observed in healthy humans, with an average of one heteroplasmic site identified per individual [7,8]. The number of heteroplasmic sites has also been shown to increase with age [7], implicating heteroplasmy in ageing phenotypes [9].

Heteroplasmy allele frequencies can change dramatically between generations. During oogenesis, there is a reduction in the amount of mtDNA (and/or in the number of mtDNA segregating units—it was suggested that mtDNA is transmitted in the form of nucleoids containing approximately 1–10 mtDNA molecules each [10–13]) inherited from the mother, leading to variable levels of the mutant and wild-type molecules passed to each offspring. Random genetic drift is the primary evolutionary force behind this germline bottleneck. Because drift is a random process, deleterious heteroplasmies that are at a low frequency in the asymptomatic carrier mother may increase in frequency in the child and reach the threshold for clinical symptoms of mtDNA disease [7].

Random genetic drift can also change heteroplasmy allele frequencies within the lifetime of an individual. First, the mtDNA genetic variants are randomly partitioned between the two daughter cells during each mitosis, leading to changes in allele frequencies among cells, organs and tissues [8]. Additionally, it has been suggested that a bottleneck may occur during embryonic development of somatic tissues [8].

**Figure 1.**
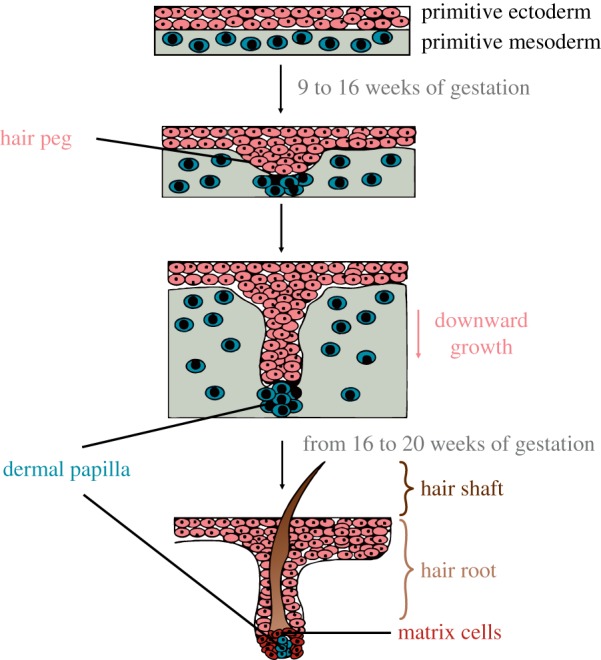
The embryological development of hair. Overview of the different steps of embryological hair development shown for a single hair. At 9–12 weeks of gestation, a discrete group of cells from the primitive ectoderm (red cells) gathers and begins to grow downward (forming the hair peg). In the primitive mesoderm (blue cells), another group of cells gathers, meeting the downgrowing ectodermal cells. This group of mesodermal cells will later form the dermal papilla as well as the fibrous sheath of the follicle. The fetal hair peg further enlarges and forms a bulbous mass that encloses the primitive dermal papilla (hair bulb). This whole arrangement of cells is also called hair follicle and produces the hair. The dermal papilla influences the hair forming matrix cells (dark red) in the hair bulb through growth factor secretion, but does not directly contribute cells to the hair shaft. By 16–20 weeks of gestation, all hair follicles are formed and begin producing hair.

The somatic mtDNA bottleneck during the development of human hair is of particular interest because of applications of this sample type in forensics. Unlike many other tissues (e.g. blood [14]), hairs develop from a small number of cells. To better understand the somatic bottleneck in hair, we first briefly review hair embryology [15] (figure 1). Hair has primarily ectodermal origins, with some contribution of the mesodermal germ layer to the dermal papilla. At approximately nine to 12 weeks of gestation, a group of cells from the ectoderm begin to grow downward to form a hair peg, which meets a group of mesoderm cells that will later form the dermal papilla and the sheath of the follicle. The dermal papilla functions to influence the matrix cells in the hair bulb by directing the mitotic activity. By week 16–20 of gestation, the hair follicles (consisting of the cell groups described above) are established and begin producing hairs. This set of follicles, which averages around 5 million, developed prior to birth is all an individual will have during his/her lifetime [15]. Each root of the hair is formed from a small group of stem cells in the hair follicle [16]. This leads to a limited pool of mtDNA molecules, i.e. somatic bottleneck [15]. Developing hair follicles give rise to hair root (portion of the hair within the described cell layers) and shaft (portion of the hair grown out of the described cell layers) via rapid mitotic cell divisions, with each division leading to a stochastic segregation of mtDNA and thus additional genetic drift [16]. The cells forming the hair shaft differentiate and never divide again.

Analysing mtDNA, instead of nuclear DNA, is useful in forensics because of its relatively high copy number in tissues such as hair shafts, old bones and teeth (samples containing a low amount of intact nuclear DNA) [17]. Additionally, owing to its high copy number and circular nature, mtDNA is less prone to degradation than nuclear DNA. Furthermore, mtDNA is highly variable allowing for an enhanced identification of individuals [18]. Moreover, because mtDNA is maternally inherited, the sample can be compared to reference samples from maternal relatives. The presence of heteroplasmy may complicate the analysis of mtDNA; however, when interpreted correctly, it presents an advantage. Although heteroplasmy allele frequencies are not used to verify the identification of samples owing to the possible variation between tissue types, they are used to increase the strength of the identification [19]. For example, if heteroplasmy is detected at a specific site in the sample, and the suspect exhibits similar levels of this heteroplasmy, the evidence is strengthened.

In this study, we explored the somatic bottleneck and changes in heteroplasmy allele frequencies occurring in human hair. The mtDNA from multiple hairs collected for each of 11 different individuals was extracted, amplified and sequenced using next-generation sequencing technology in order to analyse the allele frequencies at sites previously identified to be heteroplasmic in blood and cheek of the same individuals [20]. Multiple hairs from each individual were analysed in order to study the somatic bottleneck each hair undergoes. Eighteen hairs were separated into hair shaft and root segments to determine whether there was a difference in heteroplasmy allele frequency between these two distinct anatomical regions of hair. As a result, we determined the size of somatic mtDNA bottleneck in hair and analysed the changes in heteroplasmy allele frequency as related to human ageing. Our results contribute to our understanding of mtDNA variation in the human body and have implications for forensics.

## 2. Methods

### (a) Sample selection and DNA extraction

Hair samples were collected from mothers and their children (under IRB 30432EP) in Central Pennsylvania. Hairs from 11 individuals were selected for DNA extraction based on the number of hairs available and the frequency of heteroplasmic alleles observed in their blood and cheek tissue samples (electronic supplementary material, note S1). Only hair samples from individuals who had previously been determined to have a heteroplasmy with a minor allele frequency (MAF) greater than 5% in their blood and cheek samples were included to increase our chances of finding a heteroplasmy in the hair [20]. DNA from five to 10 single hairs was extracted from each individual, with a total of 106 hairs included in the study initially and 87 hairs (with two to eight per person) remaining after quality control of sequence data (see §2d, mtDNA mapping and variant calling). Before starting the extraction, the hair was examined to determine whether the root was present. The root was available for 18 of the selected hairs, belonging to three separate individuals (four to eight roots per person). For these hairs, one extraction was performed on the root region of the hair, and a second extraction for the shaft region. Extraction and isolation of the DNA from the hair particle was performed using the Qiagen DNeasy Blood & Tissue Kit. DNA was isolated from each hair combining two pieces from the individual hair with a size of 1–1.5 cm each; however, for very thin hairs, the amount was adjusted (a third piece of 1–1.5 cm was added) to ensure that a sufficient amount of DNA was isolated. DNA was eluted in a volume of 100 µl.

### (b) DNA amplification for sequencing

Prior to amplification of hair mtDNA for sequencing, the mtDNA copy number in the hair samples was quantified using real-time polymerase chain reaction (PCR) (BioRad CFX96 Touch System). This was important to ensure that a sufficient number of mtDNA copies was used as a template for the subsequent PCR, allowing the detection of heteroplasmy frequencies greater than or equal to 1% (our detection limit with this next generation sequencing-based approach). PCR reactions contained 1 µl isolated DNA, 5 µl 2× PowerUp™ SYBR™ Green Master Mix (Thermo Fisher Scientific), 3.6 µl PCR-grade water and 0.2 µM each primer specific for human mtDNA (F: CCACAGCACCAATCCTACCT, R: GTCAGGGGTTGAGGTCTTGG, amplifying positions 14 359–14 425 of the revised Cambridge reference sequence (rCRS)). A standard (10^6^, 10^5^, 10^4^, 10^3^, 10^2^ molecules of a plasmid containing the target mtDNA region) was used to quantify the mtDNA copy numbers in each sample. mtDNA copy number for each sample was quantified in triplicates. No template controls were included in each experiment. Amplification was performed using the following protocol: 95°C for 2 min, followed by 45 cycles at 95°C for 15 s, 57°C for 20 s, 72°C for 30 s, and following the completion of these cycles 72°C for 2 min. Melting curve analysis ensured correct amplification. Samples with less than 375 copies µl^−1^ (approx. 6500 mtDNA template molecules in the PCR) were excluded—this (larger) number was chosen to avoid bias from DNA fragmentation that can lead to lower numbers of effectively amplified mtDNA molecules in the subsequent amplifications for sequencing than measured in the quantification PCR.

To analyse the heteroplasmies present in the mtDNA, the samples were first amplified. Primers (electronic supplementary material, table S1) were designed to amplify 401–767 bp regions containing the heteroplasmic sites in the mtDNA. PCR of the samples was conducted using a 17.5 µl aliquot of the isolated DNA, which was added to 25 µl of Q5 High-Fidelity 2× Master Mix (NEB)**,** 2.5 µl of 20× EvaGreen^®^ DNA-binding Dye (Biotium) and 2.5 µl of each specified 10 µM primer for the sample. PCR was conducted on a real-time PCR thermal cycler with the following protocol: 98°C for 30 s, followed by 45 cycles at 98°C for 10 s, Tm (see the electronic supplementary material, table S1) for 1 min, 65°C for 15 s, and following these cycles 65°C for 5 min. Melting curve analysis ensured correct amplification.

### (c) Library preparation and sequencing

Libraries were prepared for Illumina sequencing. From the 106 hairs used in the extractions, libraries were prepared for 115 samples because several individuals contained more than one heteroplasmic site of interest. Gel electrophoresis (on a 1% agarose gel) was performed for all samples and band intensities were quantified using the BioRad Quantity One software, to estimate the ratios of samples needed for approximately equimolar pools. The 115 PCR products were combined into 24 pools (electronic supplementary material, table S2), with each pool containing four to six samples (PCR products of different regions in the mtDNA).

To control for cross-sample contamination during library preparation, DNA spike-ins were added to the pools: no spike-in, PhiX RF I DNA (NEB) and pUC18 plasmid DNA (Thermo Fisher Scientific) were added in alternating order (as previously described in [7]). Prior to addition, spike-in DNA was fragmented to approximately 550 bp using the BioRuptor (Diagenode): approximately 100 ng in 100 µl of PhiX RF I DNA or pUC18 plasmid DNA were fragmented with the low power setting. The cycle conditions were set to on for 30 s, followed by off for 90 s for six cycles.

The libraries were purified with 0.8 volumes of AMPure XP beads (Beckman Coulter). The concentration of the purified samples was measured using the Qubit™ Broad Range Assay to determine the amount of spike-in to be added to each pool to result in a final volume of 50 µl.

Initial library preparation steps of the 24 pools for sequencing were performed with the NEBNext Ultra II DNA Library Prep Kit for Illumina (NEB; according to manufacturer's instructions) starting with 2.5 µg of each of the 24 pools (PCR products pooled in equimolar proportions). A modification was made in the adaptor ligation step of the protocol: adapters were used from the TruSeq DNA Single Indexes Set A and TruSeq DNA Single Indexes Set B kits (Illumina). Approximate concentrations of the libraries were determined using the Qubit™ HS Assay Kit. Purity of the libraries was controlled on a Bioanalyzer High Sensitivity DNA Assay. A final library quantification was performed using the real-time thermal cycler using the KAPA Library Quantification Kit (KAPA Biosystems). Paired-end sequencing on the MiSeq instrument was performed using the MiSeq v.2 Reagent Kit (500 cycles) for Illumina.

### (d) mtDNA mapping and variant calling

The sequencing reads were analysed and filtered using Galaxy [21]. A workflow was created to filter the results, to view the sequenced sites, and the observed heteroplasmic frequencies at these sites. Quality of the sequencing reads was checked using FastQC [22]. Reads were mapped to the human reference genome (hg38) using BWA-MEM [23]. BAM files were filtered (i) to have a mapping quality of at least 20, (ii) for primary alignment, (iii) for proper pairing of pair-end reads, (iv) to map the mate, and (v) for reads aligning to the rCRS. Bam Left Align was then used to re-align the positional distribution of insertions and deletions present in the sequence. The Naive Variant Caller (NVC) tool [24] was used to call variants using a minimum number of reads needed to consider a reference allele/alternative allele equal to 10, minimum base quality of 25, minimum mapping quality of 40 and ploidy of 1. Variants were annotated using the Variant Annotator tool on Galaxy [21]: frequency threshold of 0 and a coverage threshold of 10. From the data produced, samples with a sequencing depth greater than 1000× at the heteroplasmic site were determined to be suitable for the analysis. Owing to the previous knowledge of the positions of the heteroplasmic sites (from sequencing of blood and cheek samples), in some instances, we included samples with a lower depth. Samples with a sequencing depth less than 1000× at the heteroplasmic site were included if the depth of a minor allele was greater than or equal to 9× (unlikely to be the result of sequencing errors), allowing a reliable measurement of the MAF of these heteroplasmic sites. Ten samples were excluded from further analysis because they did not meet these criteria. The average sequencing depth across all sites was 7807× (74–39 314) and across sites with depth less than 1000× was 498×. In fact, all but one site (in a single individual) had depth greater than 200×. This single site had depth of 74× and resulted in a MAF of approximately 16%; we included this data point because at this site we observed MAF of 41% in cheek and 47% in blood, and thus we expected (and observed) relatively high MAF also in hair. A MAF of 1% was used as the cut off for a site to be considered heteroplasmic, to avoid bias coming from PCR and sequencing errors. The Galaxy workflow is shown in the electronic supplementary material, figure S1. The locations of heteroplasmic sites in the mtDNA and the resulting amino acid changes were also explored to determine whether the variants were associated with any diseases [25–27].

### (e) Population genetic and statistical analysis

We estimated the effective size of the somatic bottleneck that occurs in hair development: we used the approach developed by Millar [28] which they used to estimate the germline bottleneck in penguins, with several modifications. The following equation was used to perform this calculation:

$$N_{ij}=\frac{\;p_{ij}(1-p_{ij})}{\sigma_{\mathrm{hair}}^{2}},$$

where *N_ij_* is the bottleneck size and *p_ij_* is the mean allele frequency between blood and cheek tissues for the *i*th individual and *j*th position. The mean squared deviation across *n_ij_* hairs for the same individual and position is calculated as $\sigma_{\mathrm{hair}}^{2}=\sum_{k=1}^{n_{ij}}{\left(p_{ijk}^{\mathrm{hair}}-p_{ij}\right)}^{2}/n_{ij}$, where $p_{ijk}^{\mathrm{hair}}$ is the allele frequency for the *k*th hair. We obtained the mean bottleneck size by taking the mean across *N_ij_*. Bootstrapped confidence intervals (CIs) for the somatic bottleneck were generated by sampling values of *N_ij_* with replacement 1000 times.

We also calculated pairwise divergences in heteroplasmy frequency (hereafter abbreviated simply to ‘divergence’) between blood, cheek and hair tissues. For each pair, we first calculated Hudson's *F_ST_*, and then the divergence as $d_{xy}=-2\times \text{log}(1-F_{ST})$ in *R*. Hudson's *F_ST_* was calculated using

$${\hat{F}}_{ST}^{\mathrm{Hudson}}=\frac{{\left(p_{1}-p_{2}\right)}^{2}-(p_{1}(1-p_{1})/(n_{1}-1))-(p_{2}(1-p_{2})/(n_{2}-1))}{\;p_{1}(1-p_{2})+p_{2}(1-p_{1})},$$

where *p*_1_ is the heteroplasmy frequency in tissue 1, *p*_2_ is the heteroplasmy frequency in tissue 2, and *n*_1_ and *p*_2_ represent the number of mtDNA molecules samples from tissue 1 and 2, respectively. This function was applied to each heteroplasmic site separately to get a single estimate of divergence for each heteroplasmy. We assumed *n_1_* = *n_2_* = 1000 as we cannot determine this information in droplet digital PCR data.

The relative branch length leading up to each leaf was calculated using the divergence estimates from all pairs of comparisons. If A represents blood, B represents cheek and C represents hair, we can estimate the branch leading up to A with the formula (*d*_AC_ + *d*_AB_ − *d*_BC_)/2, the branch leading up to B with (*d*_AB_ + *d*_BC_ − *d*_AC_)/2 and the branch leading up to C with (*d*_BC_ + *d*_AC_ − *d*_AB_)/2, where *d_XY_* represents the divergence in heteroplasmy frequency between tissues *X* and *Y*.

### (f) Likelihood model of sequential bottlenecks

**Figure 2.**
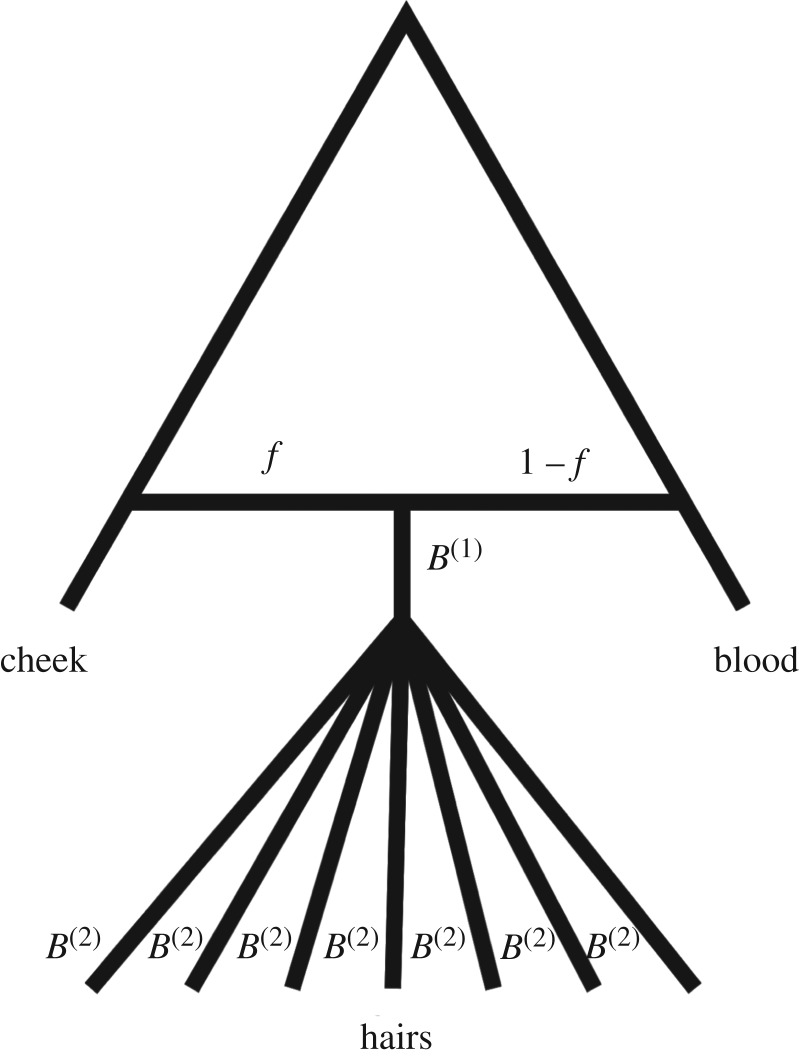
Schematic of likelihood model of mtDNA lineage development in individual hairs. *f*, *B*^(1)^ and *B*^(2)^ correspond to the quantities defined in the text.

We further explored mtDNA bottleneck dynamics during hair development by analysing a likelihood model that accounted for the possibly admixed ancestry of hair (with respect to ectodermal and mesodermal germ layers, as represented by cheek and blood respectively) and bottlenecks before and after the mtDNA lineages of individual hair follicles diverge (figure 2). Specifically, in our model, for each individual *i*, the heteroplasmy frequency *a_i_* at the establishment of the mtDNA lineage ancestral to all hair follicles is modelled as the mixture

$$a_{i}=fb_{i}+(1-f)c_{i},$$

where *f* is the admixture fraction (0 ≤ *f* ≤ 1), *b_i_* is the measured heteroplasmy allele frequency in individual *i*'s blood sample and *c_i_* is the corresponding allele frequency in the cheek sample. Given *a_i_*, the model assumes that the mtDNA lineage ancestral to all sampled hairs undergoes a binomial bottleneck of size *N_1_* such that the frequency just prior to the divergence of all hairs for individual *i* is $B_{i}^{\left(1\right)}/N_{1}$, where

$$B_{i}^{\left(1\right)}∼\mathrm{binomial}\;(N_{1},a_{i}).$$

Then, for each hair indexed *j* sampled from individual *i*, the mtDNA lineage ancestral to hair *j* undergoes an independent binomial bottleneck of size *N*_2_, producing $B_{i}^{\left(2\right)}$ mutant mtDNA copies and $N_{2}-B_{i}^{\left(2\right)}$ non-mutant mtDNA copies such that

$$B_{i}^{\left(2\right)}|B_{i}^{\left(1\right)}∼\text{binomial}\;\left(N_{2},\frac{B_{i}^{\left(1\right)}}{N_{1}}\right).$$

Finally, the observed heteroplasmy allele frequency *h_ij_* is assumed to be produced by an expansion of the $B_{i}^{\left(2\right)}$ and $N_{2}-B_{i}^{\left(2\right)}$ mutant and non-mutant mtDNA copies up to an infinitely large population size according to replication dynamics in which, for an infinite number of duplications, a single mtDNA genome is chosen uniformly at random to duplicate itself from the current pool of mtDNA haplotypes. This type of expansion is equivalent to a Pólya urn scheme, for which the limiting distribution of the frequency of mutant mtDNA copies becomes a beta distribution as the number of duplications goes to infinity [29]:

$$h_{ij}|B_{i}^{\left(2\right)}∼\text{beta}\;(B_{i}^{\left(2\right)},N_{2}-B_{i}^{\left(2\right)}).$$

Thus the likelihood equation for parameters *θ* = (*f*, *N*_1_, *N*_2_) is

$$\begin{array}{rl} L(\theta |h) & =Pr(h|\theta )\\ & =\prod_{i=1}^{n}\sum_{k=0}^{N_{1}}\psi (k;N_{1},a_{i})\prod_{\;j=1}^{\left|h_{i}\right|}\sum_{l=0}^{N_{2}}\psi \left(l;N_{2},\frac{k}{N_{1}}\right)\gamma (h_{ij};l,N_{2}-l), \end{array}$$

where ***h*** is the collection of all hair frequencies across individuals, |*h_i_*| is the number of hairs for individual *i*,

$$\psi (k;n,p)=\left(\begin{array}{c} n\\ p \end{array}\right)p^{k}{\left(1-p\right)}^{n-k}$$

is the probability mass function of a binomial distribution with parameters *n* and *p*, and

$$\gamma (x,a,b)=\frac{x^{a-1}{\left(1-x\right)}^{b-1}}{\beta (a,b)}$$

is the probability density function of a beta distribution with parameters *a* and *b*. We assume that *γ* (*x*, *a*, *b*) = 1 when *a* = 0 or *a* = *b*, which enforces no additional genetic drift if the allele fixes or is lost during the second bottleneck. For a visual representation of this model, see figure 2.

We perform inference in a Bayesian context where the marginal prior distribution for *f* is uniform between 0 and 1, the marginal prior distributions for *N*_1_ and *N*_2_ are uniform between 2 and 500, and the joint prior distribution is the product of the marginal prior distributions. To calculate posterior distributions, we used ensemble Markov chain Monte Carlo as implemented in the Python module emcee [30]. We ran an ensemble of 100 chains for 20 000 iterations, which quickly achieved convergence as assessed by visual inspection. Conservatively, we discarded the first 4000 iterations as burn-in. All likelihood calculations were performed in log-space using Python's numpy, scipy and mpmath modules. We note that the final sum in the likelihood equation above can be simplified to speed up calculations using the following identity:

$$\begin{array}{rl} & \sum_{l=0}^{n}\psi (l;n,p)\gamma (h;l,n-l)=\\ & n(n-1)p(1-p){\left[\left(h-1)(p-1\right)\right]}^{n-2}{}_{2}F_{1}\left(1-n,2-n,2,\frac{hp}{\left(h-1)(p-1\right)}\right), \end{array}$$

where _2_*F*_1_(*a*, *b*, *c*, *z*) is the ordinary hypergeometric function.

We also note that bottleneck size estimates in this model will be larger than our variance-based estimates above owing to the additional genetic drift in this model during the expansion of the mtDNA population up to a large size following the second bottleneck. In addition to making this model more realistic, this expansion is necessary because the observed frequencies take on continuous values between 0 and 1 and the binomial frequencies take on only a finite number of discrete values.

## 3. Results

### (a) Samples and sequence analysis

We collected five to 10 hairs from 11 individuals to analyse the heteroplasmy allele frequency in hair at nine specific mtDNA positions, previously determined to be heteroplasmic in blood and cheek samples from the same individuals [20]. Based on the data for the blood and cheek from the same individuals, nine individuals were heteroplasmic at one mtDNA position each, one individual (m188c2)—at two positions, and another individual (m163c1)—at three positions (table 1). Some heteroplasmic positions were shared among individuals because our sample included two mother-child pairs (m137 and m137c1; m166 and m166c5) and one grandmother-grandchildren trio (m164g, m163c1 and m163c2; table 1).

**Table 1.** Summary table of the sequenced samples. (Allele frequencies for the 14 sites sequenced in the blood, cheek and hair samples of 11 individuals. The MAF of the hair sample listed in the table is an average of the MAF of all shafts collected from the donor. The designation of minor versus major allele is based on the minor allele found in the blood and cheek tissue. For those sites located in the coding region of the mtDNA, the base change from the minor allele and resulting amino acid were recorded to determine if it was a synonymous or non-synonymous heteroplasmy.)

ID	age	position	major/minor allele	MAF blood	MAF cheek	MAF shaft/ root	hair variance shaft/root	blood and cheek variance	total hairs	shafts/roots	region	amino acid change
m137	47	16 320	C/T	0.052	0.264	0.283	0.154	0.084	8	8/0	D-loop	—
m137c1	26	16 320	C/T	0.052	0.187	0.066	0.07	0.043	5	0/5	D-loop	—
m164g	78	12 192	G/A	0.407	0.593	0.242	0.729	0.034	4	4/0	tRNA	—
m163c1	24	12 192	G/A	0.44	0.44	0.246	0.321	0.000	8	8/0	tRNA	—
m163c2	23	12 192	A/G	0.482	0.46	0.608	0.104	0.001	6	6/0	tRNA	—
m163c2	23	12 358	A/T	0.063	0.059	0.009	0.017	0.000	6	6/0	MT-ND5	non-synonymous
m163c2	23	13 842	A/C	0.151	0.153	0.11	0.02	0.000	4	4/0	MT-ND5	synonymous
m166	39	1585	A/G	0.059	0.054	0.064/0.005	0.183/0.005	0.000	8	8/6	rRNA	—
m166c5	15	1585	A/G	0.195	0.215	0.183/0.337	0.270/0.227	0.001	4	4/4	rRNA	—
m183	51	709	A/G	0.239	0.232	0.674	0.481	0.000	2	2/0	rRNA	—
m186c1	21	13 951	T/C	0.057	0.054	0.014	0.046	0.000	8	8/0	MT-ND5	non-synonymous
m188	56	5107	C/T	0.136	0.126	0.156/0.179	0.376/0.474	0.000	8	8/8	MT-ND2	non-synonymous
m188c2	17	5107	C/T	0.172	0.195	0.325	0.121	0.001	8	8/0	MT-ND2	non-synonymous
m188c2	17	16 240	A/G	0.067	0.107	0.053	0.026	0.005	8	8/0	D-loop	—

mtDNA from individual human hairs was amplified, sequenced using Illumina paired-end read sequencing, and the sequencing reads were mapped to human reference mtDNA sequence (see Methods). The obtained mean sequencing depth across all heteroplasmic sites was 7807× (ranging from 74 to 39 314). After filtering based on sequencing depth and other quality control criteria (see Methods), we retained 105 heteroplasmic sites in 87 individual hairs (table 1; electronic supplementary material, table S3). Thus, we retained data on two to eight hairs per individual. Allele frequency at site 12 192 in individual m163c1 was validated with Sanger sequencing, and the two methods (Illumina and Sanger sequencing) led to very similar results (frequency of heteroplasmy in hair 2 of 0.819 and 0.793, respectively). Our previous study [7] has also demonstrated high correlation in heteroplasmy allele frequency between Illumina and Sanger sequencing. The distinction between minor versus major allele was made based on heteroplasmy allele frequency in blood and cheek.

We next examined the location of the nine heteroplasmic positions in the mtDNA (table 1). Positions 709 and 1585 were located in a region coding for ribosomal RNA (rRNA); position 12 192 was located in a region coding for transfer RNA (tRNA); and positions 16 240 and 16 320 were located in the D-loop. The remaining four heteroplasmic positions were found in the protein-coding regions: position 5107 in *ND2*, and positions 12 358, 13 842 and 13 951—in *ND5*. Heteroplasmy at site 13 842 was synonymous, while heteroplasmies at sites 5107, 12 358 and 13 951 were non-synonymous. None of the heteroplasmic alleles was associated with any known disease-causing variants [25–27].

### (b) Variance of heteroplasmy minor allele frequencies among hairs

**Figure 3.**
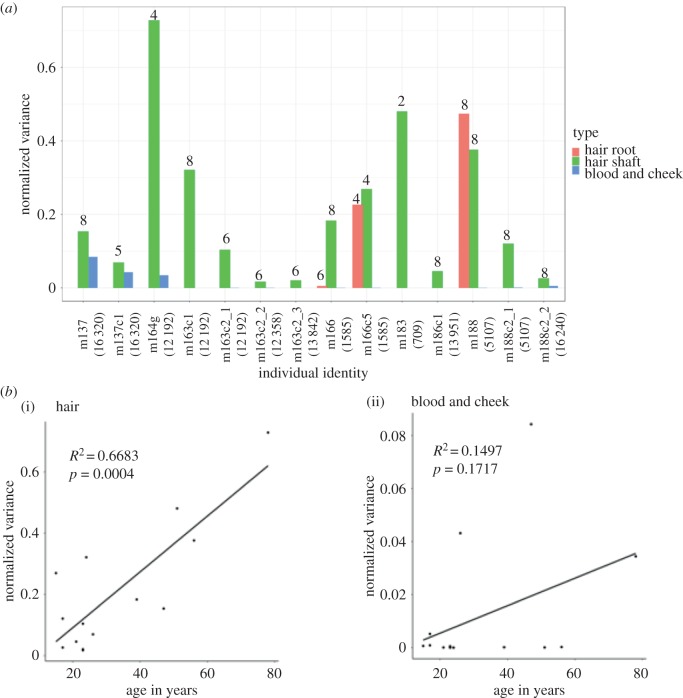
Variance in minor allele frequency (MAF) for hair, blood and cheek. (*a*) Normalized variance of heteroplasmy MAFs in hairs (shaft was measured for all donors and sites, root was measured for three donors) and other tissues (blood and cheek) collected from the same donor. Multiple positions heteroplasmic in the same donor are listed separately. The number above each column indicates the number of hair shafts or hair roots included in the analysis for each individual. Normalized variance in heteroplasmy MAF of blood and cheek was calculated for all individuals for comparison, and was very low for most samples (therefore, no bar is visible). (*b*) The correlation between donor age and variance in MAF in hair shafts (i) and blood and cheek (ii), respectively.

We observed a wide range of variance in MAFs among different hairs collected from the same individual. This analysis was limited to hair shafts because they were available for all hair samples. Even though the hair shafts were collected at a similar location of the scalp from the same individual, the MAF observed at the analysed mtDNA position differed among separate hairs (electronic supplementary material, table S3). To compare the variance across heteroplasmies at different frequencies, we normalized the variance among hair samples by dividing it by *p*(1 − *p*)—the expected variance under the Wright–Fisher model, which assumes binomial sampling of alleles in every generation (see Methods). The normalized variance ranged from 0.026 to 0.729, with an average variance of 0.208 (figure 3*a*). By contrast, the normalized variance in MAF of non-hair somatic tissues ranged from 1.1 × 10^−7^ to 0.084, with an average of 0.012—much lower than that in hair shafts (figure 3*a*).

Additionally, we sometimes observed dramatic shifts in MAF between blood, cheek and hair samples. For instance, for heteroplasmic position 12 192 in individual m163c1, the frequency of the minor allele A in blood was 0.440 and in cheek was also 0.440. However, the average MAF for the hair shafts was 0.246, and for seven out of eight hair shafts frequency of allele A was below 0.440 (electronic supplementary material, table S3). In one of the hair shafts from the same individual, the frequency of the A allele was 0.819 (electronic supplementary material, table S3). These observations highlight the high variance in allele frequency among different hairs from the same individual.

We also explored the variation in MAF observed in two distinct regions of the same hair: hair root and hair shaft. While all hairs included in the study were sequenced at the shaft region, 18 hairs (belonging to three individuals, table 1) were also sequenced at the root region. Root and shaft of the same hair may have different MAF, particularly if follicular tissue is present in the root sample [15]. Because of to the growth pattern of hair, we expect the keratinized shaft to have increased variance in the MAF, because of accumulated drift from additional mitotic divisions, as the shaft grows outwards from the dividing matrix [15]. Indeed, we observed different MAFs between the shaft and root of the same hair sample (electronic supplementary material, table S3) and a higher variance in the shaft region of the hair compared to the root region for two of the three individuals (figure 3*a*). However, our sample size was very small (i.e. limited to three individuals) and thus this conclusion should be treated as preliminary. In addition, the length and thickness of a hair probably influences this analysis because the shaft region in shorter hairs is closer to the root segment, and therefore might not have diverged from it as much as in longer hairs.

Variance in MAF calculated from hairs collected from a single individual was positively correlated with the age of the donor (*R*^2^ = 0.668, *p* = 0.0004), which is in agreement with a theoretical prediction of linear mtDNA variance increase over time [31]. Thus, individual hairs of older individuals accumulated greater differences in their MAF than did hairs of younger individuals, which is consistent with cellular and mtDNA turnover events, such as cellular replication and division, that occur in hair stem cells throughout the life of an individual (figure 3*b*). This correlation was weaker and not statistically significant (*R*^2^ = 0.150, *p* = 0.172) when comparing donor age to the MAF variance in blood and cheek tissues, suggesting that the increased mitotic activity of hair compared to other tissues leads to higher variation in MAF with age in hair in particular.

### (c) Somatic bottleneck size estimation

During embryonic development and folliculogenesis, each individual hair follicle undergoes a separate genetic bottleneck [15]. Once the hair follicles are formed, this is all the individual will possess to produce scalp hair throughout their lifetime, thus the hair cells feeding the shaft with mitochondria are clonal in nature and will reflect the isolated population of mtDNA [15]. Therefore, we intended to estimate the somatic bottleneck that occurs during fetal development in each separate hair. We followed the method developed by Millar [28] for estimating the germline bottleneck in penguins, implementing minor modifications for hair samples, as described in Methods. They used maternal and offspring MAFs as pre- and post-bottleneck data, respectively. In adaptation of their method to our case (see Methods), we used mean MAF across blood and cheek samples and mean hair shafts' MAF for the same individual as pre- and post-bottleneck data, respectively. As a result, we determined the size of the somatic bottleneck in hair to be 11.06 (95% CI 0.6–34.0).

It is possible that hairs also experience some amount of genetic drift because of bottleneck(s) during embryonic development prior to the formation of individual hair follicles. We estimated the size of this bottleneck, *N*_1_, and compared it to the size of the bottleneck experienced by each hair, *N*_2_, in a maximum-likelihood framework. The median posterior size of the first bottleneck shared by all hairs (i.e. *N*_1_), was 27 (9–123; 95% highest posterior density credible interval). For the second bottleneck experienced independently by each hair (i.e. *N*_2_), the median posterior estimate was 11 (9–14; 95% CI; figure 5*b*,*c*). Thus, the bottleneck experienced by each hair is more pronounced than that shared by the hairs. If the amount of genetic drift during a bottleneck of size *N* is assumed to be proportional to 1/*N*, the fraction of genetic drift occurring before versus after the divergence of different hair follicles can be estimated using the posterior samples of $N_{1}^{-1}/(N_{1}^{-1}+N_{2}^{-1})$. Using the samples of this quantity, we estimated that 29.2% (posterior median; 4.95–48.3% 95% CI) of genetic drift in hair follicles occurs before individual hairs diverge versus after (figure 5*d*).

### (d) Comparison of heteroplasmy frequency among hair, blood and cheek

**Figure 4.**
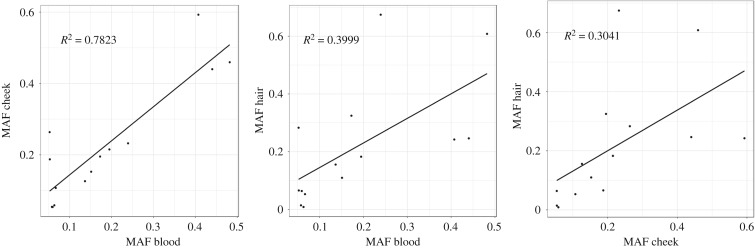
MAF comparisons between tissue pairs. The MAF found at the heteroplasmic sites in blood versus cheek, blood versus hair shafts and cheek versus hair shafts were plotted and the *R*^2^ values were calculated. The MAF for hair displayed in the figure is an average from the hair shafts collected from a single individual.

**Figure 5.**
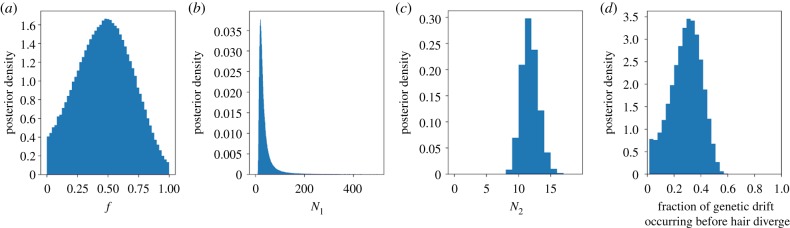
Posterior distributions of likelihood model parameters. (*a*–*c*) The posterior distributions of *f*, *N*_1_ (first bottleneck—shared by all hairs), and *N*_2_ (second bottleneck—experienced independently by each hair), respectively. (*d*) The posterior distribution of estimated fraction of genetic drift occurring before (versus after) mtDNA lineages of different hair follicles diverge. (Online version in colour.)

We explored which of the three tissues (hair, blood and cheek) are more similar to each other based on the measured MAFs within these tissues. We found that blood and cheek are more similar to each other, in terms of heteroplasmy allele frequency, than either is to hair. Using the MAFs previously determined at specific sites in blood and cheek [20] and the average MAF of the analysed hair shafts for each individual (table 1), three pairwise comparisons were performed: between blood and cheek, between hair and blood and between hair and cheek. We found that the MAF was most closely correlated between blood and cheek in our samples (*R*^2^ = 0.782, *p* = 2.66 × 10^−5^; figures 4 and 5). The correlations between the MAFs in the hair and blood samples and between the hair and cheek samples were still evident and statistically significant (hair versus blood: *R*^2^ = 0.400, *p* = 0.015; hair versus cheek: *R*^2^ = 0.304, *p* = 0.041), but were not as strong as that between blood and cheek samples.

This is consistent with the observation that the average divergence between blood and cheek (0.049) was smaller than the mean divergence between blood and hair shafts (0.152) or the mean divergence between cheek and hair shafts (0.143; electronic supplementary material, figure S2). This finding further suggests that there is more drift occurring in hair tissue since its divergence from blood and cheek lineages, probably owing to somatic bottleneck.

We also examined whether our data provide signal for admixture between ectodermal and mesodermal tissues during hair development, which is expected because of the contribution of both developmental layers to hair development [15]. We used blood as a proxy for mesoderm and cheek as a proxy for ectoderm. The calculated *f*_3_ values resulted in all but one positive values (table 2); negative values would be suggestive of admixture [32]. Therefore, we have no direct evidence of mtDNA admixture between ectodermal and mesodermal tissues during hair development. Moreover, our maximum-likelihood model of sequential somatic bottlenecks estimated the admixture fraction *f* between blood and cheek contributing to hair in the same model. The posterior median of the admixture fraction *f* between blood and cheek was 0.476 (0.0035–0.864; 95% CI), thus again suggesting that we do not have evidence to indicate that hair is more related to ectoderm versus mesoderm. An alternative explanation for this observation is common ancestry of blood and cheek samples.

**Table 2.** Calculated admixture f3 values between hair, blood and cheek. (For individuals with more than one site, the average value among sites was calculated.)

ID	site	f_3_
m137	16 320	0.004
m137c1	16 320	−0.002
m164g	12 192	0.058
m163c1	12 192	0.038
m163c2	12 192, 12 358, 13 842	0.008
m166	1585	0.000
m166c5	1585	0.000
m183	709	0.193
m186c1	13 951	0.002
m188	5107	0.001
m188c2	5107, 16 240	0.010

## 4. Discussion

### (a) High variation in mtDNA minor allele frequency among hairs of the same individual

Here, we performed a detailed experimental and population genetic analysis of MAF variation among multiple hairs collected from the same individual, and considering several individuals. Different hairs collected from the same donor displayed a wide range of MAFs at each heteroplasmic position analysed, indicating high variability of mtDNA in hair. The variance in MAF for hairs was always higher than that for blood and cheek collected for the same individual, arguing for high levels of mtDNA variation for hair in particular. It is important to note that our study was restricted only to sites previously shown to be heteroplasmic in the studied individuals. Individual hairs might have acquired somatic *de novo* mutations at other sites, and thus we might be underestimating the overall mtDNA variation in hairs. This limitation is expected to affect neither our estimations of bottleneck sizes nor our main conclusions.

Our results corroborate the conclusions of a much older study analysing multiple hairs from one individual [16]. That study found similar proportions of mtDNA haplotypes in the blood and cheek cells, but highly variable MAF among individual hairs. While the hairs analysed in our study were collected from the same region of the scalp, Bendall and colleagues found that variation among hairs collected within a 1 cm region of the scalp was as high as among the hairs collected from different regions of the scalp [16]. Another study observed heteroplasmy in hair when the blood sample provided by the same individual was homoplasmic [18].

An important question arises as to what biological processes might explain this high variation in mtDNA MAF among individual hairs. Our results are consistent with three biological processes contributing to this high variation: (i) moderate somatic bottleneck during embryonic development of all hairs; (ii) pronounced somatic bottleneck during the development of each separate hair; and (iii) additional genetic drift resulting from mtDNA segregation during rapid mitotic cell divisions occurring in the course of hair development and growth.

### (b) Somatic bottlenecks in hair

The high variance in MAF seen among hairs from the same individual can be mostly attributed to a drastic bottleneck occurring during formation of each hair follicle. Unlike other types of cells (e.g. lymphocytes), each hair follicle is formed before weeks 16–20 of gestation from a small, discrete group of stem cells, leading to a limited pool of mtDNA molecules present in each hair [15]. We estimate the effective bottleneck from the total amount of drift experienced by heteroplasmies in each hair, to be as pronounced as 11.06 (95% CI 0.6–34.0) mtDNA segregating units. To the best of our knowledge, the somatic bottleneck experienced by separate hairs has not been estimated previously. In terms of its magnitude, the effective somatic bottleneck in separate hairs appears to be similar to the effective germline bottleneck we recently estimated to be approximately 7–10 mtDNA segregating units [20], or our previous publication estimated to be 25–32 units [7].

Our analysis also indicates the presence of genetic drift during early embryonic development, i.e. before divergence of different hairs and thus preceding the somatic bottleneck of separate hair follicles. Such earlier bottleneck is shared by all hairs of an individual. We estimate its size to be 27 (9–123; 95% highest posterior density credible interval) mtDNA segregating units—less drastic than (but not statistically significantly different from) the somatic bottleneck experienced by separate hairs, which in the same maximum-likelihood model we estimate to be 11 (9–14; 95% highest posterior density credible interval). Interestingly, this ‘shared’ hair bottleneck is still probably more pronounced than the somatic bottleneck for blood and cheek we recently calculated to be 136 (74.1–248) and 458 (104–2817), respectively [33].

### (c) Somatic segregation and age effects

In addition to these early developmental bottlenecks, the variable MAF we observed can be explained by events during an individual's lifetime that lead to additional genetic drift, specifically the rapid mitotic activity of the hair matrix, the part of the hair follicle producing the hair shaft. Following follicle development, hair roots and shafts are produced from the follicle through rapid mitotic divisions of the matrix [16]. In fact, the matrix has the highest mitotic rate of any human organ, and each round of mitosis can result in the random distribution of mtDNA molecules to the daughter cells [34]. Because hair root and shaft formation is induced through rapid mitotic divisions, these successive cellular divisions could increase the variation in MAF observed in different hairs from the same individual throughout their lifetime [16].

We observed a positive correlation between the variance in the MAF of hair samples and the age of the donors, indicating that older hair donors had accumulated more variation in the MAF of their hairs. Because hairs are produced from rapid mitotic divisions, during which mtDNA is randomly segregated from a discrete number of stem cells, it is expected that older donors would experience a greater amount of genetic drift, reflected in the increased variation of MAF among the hair samples [16]. The increased variance in mtDNA MAF in older individuals may contribute to ageing phenotypes, as the shifts in MAF may reach the threshold frequency that results in mitochondrial diseases associated with increased age. In a study performed in mice, it was shown that a depletion of mtDNA led to dysfunctional hair follicles, defects in hair shaft formation and visual hair loss, an extrinsic sign of ageing [35]. When the mice were restored with functional mitochondria, these observations were reversed.

Previous studies have suggested that there is a statistically significant increase in the number of heteroplasmies in somatic tissues, such as muscle tissue, with age [9]. We previously found a positive association between the age of individual at collection and number of point heteroplasmies in blood and cheek cells, indicating that older individuals had accumulated more mutations in their somatic tissue [7]. This phenomenon was also demonstrated in another study, which suggested a fivefold increase in the frequency of point mutations at mtDNA in brain tissue over the course of 80 years in an individual [36].

### (d) Applications to forensic analysis of hair samples

Because hair is often the evidence left behind and available for analysis at a crime scene, our findings have important implications for interpreting evidentiary results in forensics.

As suggested by the results of this study, hairs collected from the same individual, even from the same location on the scalp, might possess different MAFs at heteroplasmic sites among each other, and this variability increases with the age of individuals. Additionally, we observed that minor and major allele frequency can shift between different hairs from the same individual, making identification even more difficult. Moreover, we found that MAFs in hairs frequently differ from those in blood and cheek samples. All of these observations can lead to a false elimination of a person as a suspect. Therefore, caution should be exercised when using heteroplasmy-based evidence from hair samples in forensics.

The results of our study can be used as a guide to forensic investigations because the size of the somatic bottleneck in hair we estimated here can be used to determine the expected variation in MAF among different hairs, as well as among hairs and other tissues from the same individual. Hair and blood are two of the most common tissue types left at the crime scene, and cheek cells can be collected from suspects or from victim's relatives. However, we caution that hairs collected from two maternal relatives (unless they are identical twins) are perhaps the most problematic samples for a forensic investigation employing data on heteroplasmy allele frequency. This is because each hair undergoes its own severe somatic bottleneck, augmented by the drastic germline bottleneck(s) between maternal relatives.

## 5. Conclusion

Our findings have important implications for understanding mtDNA variation among different tissues in the human body. We demonstrate that it is shaped by biological processes occurring during embryonic development and throughout a life course. While the mtDNA positions we studied were not associated with any known mitochondrial diseases, future studies should address a possibility of selection operating at mtDNA in hair. Selection acting at mtDNA was suggested to be important during the germline development [20,37], but also in the process of ageing [38]. Finally, our results have important implications in forensics. When comparing a suspect to a sample left at the crime scene, investigators must keep in mind that the heteroplasmic frequency may vary between hairs, and that a heteroplasmy may be present in the hair sample that is not present in another tissue, of the same individual.

## Supplementary Material

Supplementary notes, tables and figure

## Data accessibility

The sequencing reads are available at SRA under accession PRJNA534307. The code for population genetic analysis can be found in the electronic supplementary material, note S2 and at https://github.com/ammodramus/hair.
