# Supplementary material for: Pronounced somatic bottleneck in mitochondrial DNA of human hair
Source: Philos Trans R Soc Lond B Biol Sci. 2019 Dec 2;375(1790):20190175. doi: 10.1098/rstb.2019.0175 (PMC6939377; doi:10.1098/rstb.2019.0175)

SUPPLEMENTARY MATERIALS

Pronounced Somatic Bottleneck in Mitochondrial DNA of Human Hair

Alison Barrett*[1], Barbara Arbeithuber*[1], Arslan Zaidi[1,2], Peter Wilton[3], Ian M. Paul[4], Rasmus Nielsen[3], and Kateryna D. Makova[1#]

*These authors contributed equally

[1]Department of Biology, Penn State University, University Park, PA
[2]Current address: Department of Genetics, University of Pennsylvania, Perelman School of Medicine, Philadelphia, PA
[3]Department of Integrative Biology, University of California at Berkeley, Berkeley, CA
[4]Department of Pediatrics, Penn State College of Medicine, Hershey, PA
[#]To whom correspondence should be addressed at kdm16@psu.edu

Supplementary notes

Note S1. Identification of heteroplasmies
As part of a different project (Zaidi *et al.* in review), we sequenced the mitochondrial genomes of blood and buccal tissue samples of 345 individuals from 96 families. This was done through paired-end sequencing on an Illumina Miseq instrument. After extensive quality control, we identified 668 sites where the minor allele frequency (MAF) was greater than or equal to 0.01. To enrich for putative germline heteroplasmies, we selected sites where the MAF>0.05 in both blood and buccal tissue of the same individual. This was done to increase the probability of detecting heteroplasmies in the hair tissue from the same individual. We retained individuals for whom we had at least 2 or more hair samples, resulting in 14 heteroplasmies in 11 individuals.

References

Zaidi AA, Wilton P, Su MS-W, Paul IM, Arbeithuber B, Anthony K, et al. The germline bottleneck, maternal age, and selection modulate the transmission dynamics of mitochondrial DNA in human pedigrees. (in review).

Note S2. Analysis of heteroplasmy frequency divergence among hair, blood, and cheek

AAZaidi

2/28/2019

Note: The code used here was written for our other heteroplasmy paper (Zaidi et al.)

Load libraries and read in heteroplasmy frequency data.

```
library(ggplot2)
library(dplyr)
```

```
##
## Attaching package: 'dplyr'
## The following objects are masked from 'package:stats':
##
##     filter, lag
## The following objects are masked from 'package:base':
##
##     intersect, setdiff, setequal, union
```

```
library(data.table)
```

```
##
## Attaching package: 'data.table'
## The following objects are masked from 'package:dplyr':
##
##     between, first, last
```

```
adat<-fread("al_data.txt",header=T,sep="\t")
```

Define function to calculate Hudson's Fst for a pair of populations/tissues

```
#function to calculate the divergence b/w populations
#first calculate Fst, then dxy as -2*log(1-Fst)
#fst formulae from Bahatia et al. 2013 Gen. Research
cal_fst<-function(p1,p2,n1=1000,n2=1000){
  #p1 is allele frequency in pop1
  #p2 is allele frequency in pop2
  #n1 is sample size of pop1 (defaults to 1000)
  #n2 is sample size of pop2 (defaults to 1000)
  num=(p1-p2)^2 - ( (p1*(1-p1))/(n1-1) + (p2*(1-p2))/(n2-1) )
  den=(p1*(1-p2)) + (p2*(1-p1))

  if(den==0){fst=0}else{
    fst=num/den
  }
  if(fst==1){
    d= -2*log(1-0.99)}else{
      d= -2*log(1-fst)}
```

```
  return(c(fst=fst,dxy=d,num=num,den=den))

}
```

Apply this function to each separately for each tissue pair combination.

```
bvc<-as.data.table(t(mapply(cal_fst,adat$blood,adat$cheek)))
bvh<-as.data.table(t(mapply(cal_fst,adat$blood,adat$hair)))
cvh<-as.data.table(t(mapply(cal_fst,adat$cheek,adat$hair)))
```

Now bootstrap heteroplasmies (with replacement), calculate average Fst across heteroplasmies, and generate distribution of Dxy for each tissue pair combination.

```
#define function to bootstrap
boot<-function(x){
  ix<-sample(nrow(x),nrow(x),replace=T)
  x2<-x[ix,]
  fst1=mean(x2$fst)
  fst2=sum(x2$num)/sum(x2$den)
  dxy1=mean(x2$dxy)
  dxy2=-2*log(1-fst2)
  return(c(fst1=fst1,fst2=fst2,dxy1=dxy1,dxy2=dxy2))
}

#create matrix to store results in
boot.bvc<-matrix(NA,nrow=100,ncol=4)
boot.bvh<-matrix(NA,nrow=100,ncol=4)
boot.cvh<-matrix(NA,nrow=100,ncol=4)

for(i in 1:100){
  boot.bvc[i,]<-boot(bvc)
  boot.bvh[i,]<-boot(bvh)
  boot.cvh[i,]<-boot(cvh)
}

colnames(boot.bvc)<-colnames(boot.bvh)<-colnames(boot.cvh)<-c("fst1","fst2","dxy1","dxy2")

boot.bvc<-as.data.frame(boot.bvc)
boot.bvh<-as.data.frame(boot.bvh)
boot.cvh<-as.data.frame(boot.cvh)

boot.bvc$tissue_pair<-"bvc"
boot.bvh$tissue_pair<-"bvh"
boot.cvh$tissue_pair<-"cvh"

boot.all<-rbind(boot.bvc,boot.bvh,boot.cvh)

ggplot(boot.all,aes(tissue_pair,dxy2))+
  geom_boxplot(aes(fill=tissue_pair))+
  theme_bw()+
  theme(legend.position="none",
        panel.grid.major.x = element_blank())+
  scale_x_discrete(labels=c("Blood\nvs\ncheek","Blood\nvs\nhair","Cheek\nvs\nhair"))+
  labs(x="Tissue pair",
       y="Divergence",
```

```
    fill="Tissue pair")
```

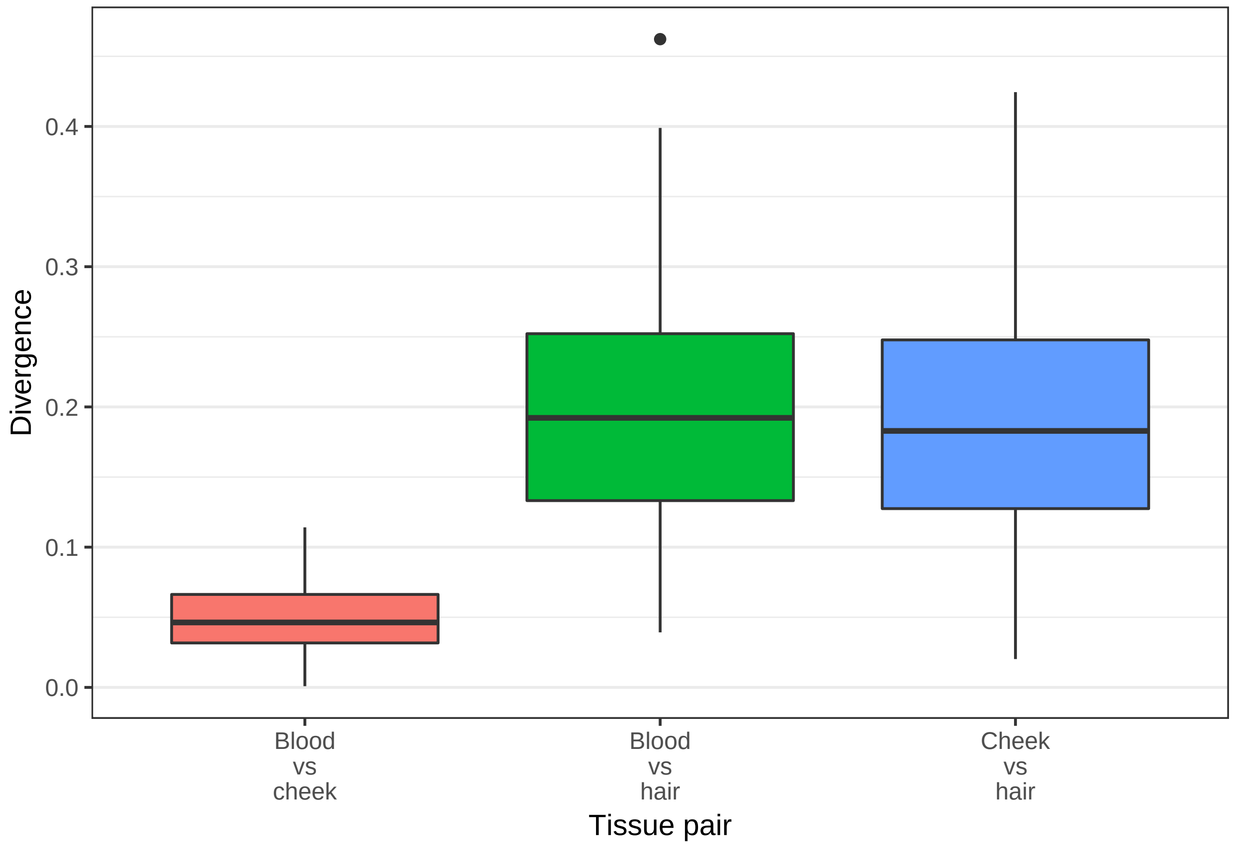

Supplementary tables

Table S1. List of primers used in amplification of DNA for sequencing

Primer Name	Sequence	Heteroplasmic site target position	PCR product length (bp)	Tm °C
**214-F**	GAGCTCTCCATGCATTTGGT	709	767	60
**214-R**	GTGTGGCTAGGCTAAGCGTTT			
**5107-F**	CTAGCCCCCATCTCAATCATA	5,107	401	59
**5107-R**	TCTTCGATAATGGCCCATT			
**13602-F**	CGCCTTCTTCAAAGCCATA	13842, 13951	650	60
**13602-R**	GGCAGGTTTTGGCTCGTAA			
**1193-F**	GCTAAGACCCAAACTGGGATT	1,585	671	60
**1193-R**	TGGTTTGGCTAAGGTTGTCTG			
**12127-F**	TCTTTGCAGGCACACTCATC	12192, 12358	478	60
**12127-R**	CTAGCCCCCATCTCAATCATA			
**15930-F**	TGGCGCCTCAATATTCTTTATC	16240, 16320	700	60
**15930-R**	CCCATACATTGGGACAGACC			

Table S2. Sample pools for sequencing. Sequencing site is included in parentheses

Pool	Sample 1	Sample 2	Sample 3	Sample 4	Sample 5	Sample 6
**1**	m183_H1 (709)	m166_1R (1585)	m188_1R (5107)	m163c1_H1 (12192)	m163c2_H1 (13842)	m188c2_H1 (16240)
**2**	m183_H2 (709)	m166_1S (1585)	m188_1S (5107)	m163c1_H2 (12192)	m163c2_H2 (13842)	m188c2_H2 (16240)
**3**	m166_2R (1585)	m188_2R (5107)	m163c1_H3 (12192)	m163c2_H3 (13842)	m137c1_H4 (16320)	
**4**	m166_2S (1585)	m188_2S (5107)	m163c1_H8 (12192)	m188c2_H3 (16240)		
**5**	m183_H3 (709)	m166_3R (1585)	m188_3R (5107)	m163c1_H10 (12192)	m137c1_H5 (16320)	
**6**	m166_3S (1585)	m188_3S (5107)	m163c1_H11 (12192)	m163c2_H4 (13842)	m137c1_H3 (16320)	
**7**	m166_4R (1585)	m188_8S (5107)	m163c1_H12 (12192)	m188c2_H4 (16240)		
**8**	m183_H4 (709)	m166_4S (1585)	m188_10R (5107)	m163c1_H13 (12192)	m137c1_H2 (16320)	
**9**	m166_5R (1585)	m188_5R (5107)	m163c2_H1 (12192/12358)	m163c2_H5 (13842)		
**10**	m166_5S (1585)	m188_5S (5107)	m163c2_H2 (12192/12358)	m188c2_H5 (16240)		
**11**	m183_H5 (709)	m166_6R (1585)	m163c2_H3 (12192/12358)	m188_6R (5107)	m188c2_H6 (16240)	
**12**	m166_6S (1585)	m188_6S (5107)	m163c2_H4 (12192/12358)	m163c2_H6 (13842)	m188c2_H7 (16240)	
**13**	m166_H7 (1585)	m188_7R (5107)	m163c2_H5 (12192/12358)	m186c1_H1 (13951)	m188c2_H8 (16240)	
**14**	m183_H6 (709)	m166_H8 (1585)	m163c2_H6 (12192/12358)	m188_7S (5107)	m188c2_H9 (16240)	
**15**	m166_H9 (1585)	m188_8R (5107)	m164g_H1 (12192)	m188c2_H10 (16240)		
**16**	m166_H10 (1585)	m188_10S (5107)	m164g_H2 (12192)	m186c1_H2 (13951)	m137_H1 (16320)	
**17**	m166c5_1R (1585)	m188c2_H1 (5107)	m164g_H3 (12192)	m186c1_H3 (13951)	m137_H2 (16320)	
**18**	m166c5_1S (1585)	m188c2_H2 (5107)	m164g_H4 (12192)	m186c1_H4 (13951)	m137_H3 (16320)	
**19**	m166c5_2R (1585)	m188c2_H3 (5107)	m163c2_H1 (12192/12358)	m186c1_H5 (13951)	m137_H4 (16320)	
**20**	m166c5_2S (1585)	m188c2_H4 (5107)	m163c2_H2 (12192/12358)	m186c1_H6 (13951)	m137_H5 (16320)	
**21**	m166c5_3R (1585)	m188c2_H5 (5107)	m163c2_H3 (12192/12358)	m186c1_H7 (13951)	m137_H6 (16320)	
**22**	m166c5_3S (1585)	m188c2_H6 (5107)	m163c2_H4 (12192/12358)	m186c1_H8 (13951)	m137_H7 (16320)	
**23**	m166c5_4R (1585)	m188c2_H8 (5107)	m163c2_H5 (12192/12358)	m137_H8 (16320)		
**24**	m166c5_4S (1585)	m188c2_H9 (5107)	m163c2_H6 (12192/12358)	m137c1_H1 (16320)		

Table S3. Overview of analyzed samples. Heteroplasmic frequencies of blood, cheek, and individual hairs are shown.

Individual ID	FID	Position	Major Allele	Minor Allele	MAF blood	MAF cheek	Hair 1	Hair 2	Hair 3	Hair 4	Hair 5	Hair 6	Hair 7	Hair 8	MAF average hair	Raw Variance	Total hairs
**m137**	F117m1c1	16320	C	T	0.05204	0.2637	0.3659	0.0013	0.381	0.177	0.2068	0.1244	0.5883	0.4189	0.283	0.031	8
**m137c1**	F117m1c1	16320	C	T	0.05200	0.1875	0.0003	0.068	0.1773	0.0836	0				0.066	0.004	5
**m164g**	F140g1	12192	G	A	0.40722	0.5928	0.8726	0.0919	0.004	0.0006					0.242	0.134	4
**m163c1**	F140g1m1c1	12192	G	A	0.44033	0.4400	0.3531	0.8194	0.2732	0.2283	0.0014	0.0243	0.1021	0.1683	0.246	0.060	8
**m163c2**	F140g1m1c2	12192	A	G	0.48202	0.4596	0.6835	0.7452	0.8215	0.3897	0.4351	0.5735			0.608	0.025	6
**m163c2**	F140g1m1c2	12358	A	T	0.06328	0.0586	0	0.0001	0	0.0001	0.0226	0.0286			0.009	0.000	6
**m163c2**	F140g1m1c2	13842	A	C	0.15117	0.1527	0.0412	0.1622	0.1317	0.1032					0.11	0.002	4
**m166**	F142m1	1585	A	G	0.05926	0.0539	0.4097	0.0103	0.0003	0.0005	0	0.0027	0.0893	0.0386	0.064	0.017	8
**m166c5**	F142m1c3	1585	A	G	0.19474	0.2152	0.3666	0.0069	0.2392	0.1183					0.183	0.018	4
**m183**	F154m1	709	A	G	0.23932	0.2323	0.3495	0.9993							0.674	0.106	2
**m186c1**	F156m1c1	13951	T	C	0.05747	0.0541	0	0.002	0	0	0.0713	0.0408	0	0	0.014	0.001	8
**m188**	F157m1	5107	C	T	0.13648	0.1262	0.3273	0.0334	0.0124	0.3453	0.0012	0.4376	0.0142	0.0727	0.156	0.029	8
**m188c2**	F157m1c2	5107	C	T	0.17253	0.1953	0.6681	0.4029	0.262	0.2086	0.2469	0.4379	0.2657	0.1052	0.325	0.027	8
**m188c2**	F157m1c2	16240	A	G	0.06672	0.1073	0.0472	0.0029	0.0631	0.101	0.0389	0	0.1009	0.0704	0.053	0.001	8
**Roots**							**Hair 1R**	**Hair 2R**	**Hair 3R**	**Hair 4R**	**Hair 5R**	**Hair 6R**	**Hair 7R**	**Hair 8R**			
**m166**	F142m1	1585	A	G	0.05926	0.0539	0.0007	0.01	0.0007	0.0144	0.0053	0.0004			0.005	2.84E-05	6
**m166c5**	F142m1c3	1585	A	G	0.19474	0.2152	0.4732	0.0835	0.1578	0.6328					0.337	5.06E-02	4
**m188**	F157m1	5107	C	T	0.13648	0.1262	0.0005	0.0141	0.0003	0.4481	0.0004	0.7521	0.0004	0.2133	0.179	7.93E-02	8

Supplementary figures

Figure S1. Galaxy Workflow for variant calling

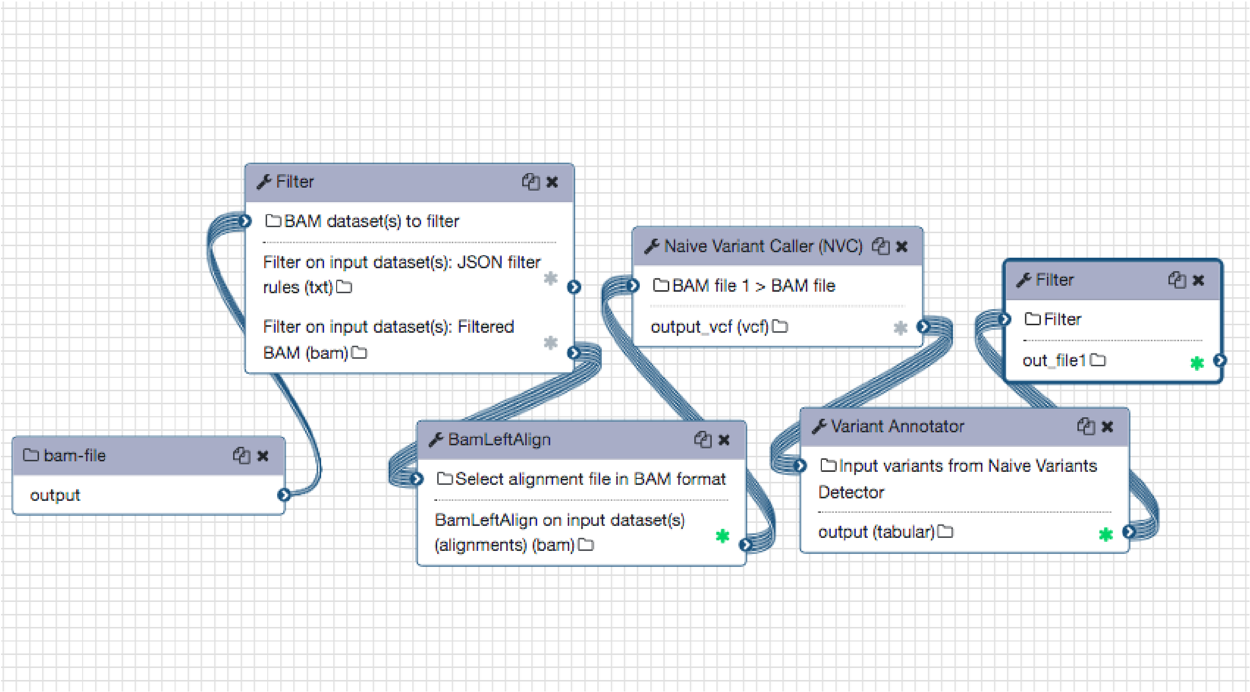

Figure S2. The relationship among blood, cheek, and hair (a schematic representation). A. Using the calculated divergences, it was determined that blood and cheek have more similar MAF. **B.** The calculated divergence estimates between blood vs. cheek, blood vs. hair, and cheek vs. hair.

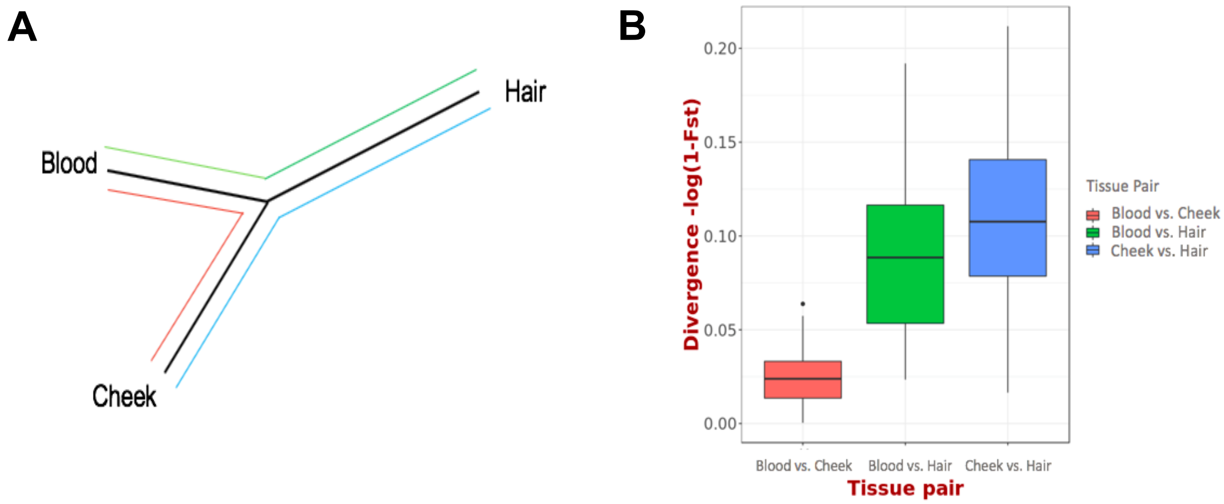

Supplement: Supplementary notes, tables and figure [file rstb20190175supp1.pdf]
